# Medical and Topographical Observations upon the Mediterranean; and upon Portugal, Spain, and Other Countries

**Published:** 1839-09-14

**Authors:** 


### BIBLIOGRAPHICAL NOTICE.

Medical and Topographical Observations *upon the Mediterranean; and upon Portugal, Spain, and other Countries.* By G. R. B. Horner, M. D., U. S. N., &c. &c. Philadelphia: Haswell, Barrington, and Haswell. 1839. 8vo., pp. 212.

These observations are the fruits of two cruises in the Mediterranean, comprising a period of six years,—the first performed in the John Adams, in 1831, '2, and '3; the second in the United States, in 1836, '7, and '8. They form a very agreeable *olla podrida* of entertaining and instructive matter, including notices of the climate, scenery, botanical and mineralogical productions, hospitals, and public institutions, of the fine countries washed by the Mediterranean.

The work opens with general observations on the climate of the Mediterranean, and the diseases which occurred in the crews of the vessels to which Dr. Horner was surgeon. The climate of the Mediterranean is temperate and humid, the character of the diseases various. Pulmonary affections, particularly phthisis, were exceedingly prevalent among the American crews. We learn that—

"During the last cruise, the number of cases of phthisis was very great, no less than eight of the men, and two of the officers having died of it, either on board the ship or on shore; save one of the latter, who died while returning home in another vessel. This number of deaths from phthisis was much disproportioned to that from other complaints, and although three of them originated in the United States; nevertheless, it serves to prove, that, notwithstanding the climate of the Mediterranean is celebrated for its mildness, and suitableness to consumptive persons, it is not as beneficial to them as is represented, and that not only they, but the well, should not think that while living in it they are out of danger, and will enjoy an exemption from this disease. Indeed, the climate, so far from being thought adapted to such patients, is believed by some persons to be decidedly injurious, and instead of putting a stop to the disease, to hurry on its progress to a fatal termination."

These views, at variance with popular impressions, are confirmed by other writers. Dr. Sinclair, of the British Navy, states, that, in the years 1810, '11, '12, out of a fleet of thirty thousand men, two thousand one hundred and thirteen of whom were on the sick list, four hundred and fifty-five were affected with phthisis pulmonalis. In a recent notice of the Statistical Report of the British West India Troops, (No. 4, p. 60,) we adverted to a similar fact with regard to the West Indies. Phthisis is developed to a greater extent, and is more rapidly fatal among the troops quartered in these islands, than among those serving in Great Britain.

There was an epidemic of cholera on board the John Adams, off Constantinople, in 1831. The non-contagious character of the disease is illustrated by the following facts:

"No person belonging to the vessel was near, or saw any inhabitant of Constantinople or of its vicinity who was, or to the best of my knowledge had been, affected with the disease. The officers were the first who went ashore, wandering through the streets, the bazaars, and other places; they mingled with crowds, formed of every class of the people, but chiefly of the lowest; which, as every where else, had suffered most from the disorder, and they yet were the last persons aboard who took it, and they were affected in the mildest manner. Their servants and the boatmen, who were similarly exposed, in like manner escaped with a few exceptions. Again, while the ship was at Long Island, the launch, with a crew of seventeen men, one of whom died of cholera, was sent frequently to Vourla for water, and though they without restraint associated with the people assembled there about the fountain, or crowding a grocery shop near it, nevertheless none of these latter were infected with the disease. Lastly, many persons of both sexes, and both young and old, belonging to the adjacent country, frequently visited the island on business or to gratify their curiosity. Some of them came near, others walked through the ancient reservoir where all the sick were placed, and notwithstanding they were so exposed to infection, none of them contracted the disorder, nor communicated it to their friends and neighbours when they returned home."

Small-pox appeared twice on board the frigate United States, during the Doctor's cruise. He carefully vaccinated the whole crew with matter obtained from the vaccine institution at New York, and failed to produce a single genuine pustule on any one, whether previously vaccinated or not. This general failure is attributed *not* to impurity of the matter, but to an insusceptibility to the virus, which, the Doctor is inclined to think, exists in hardy, robust adults. But, do not the facts here prove too much? and would not a partial failure have been more to the point?

*En passant*, Dr. Horner expresses his disbelief in the doctrine, that, after a number of years, vaccination loses its protective power: he never met with any adult, once vaccinated in childhood, who was affected with small-pox, either in its virulent or modified character. The mass of evidence, however, seems to be on the other side of the question.

From general, Dr. Horner passes to special observations upon Portugal, Spain, and other countries of the Mediterranean. Out of a somewhat heterogeneous mass of information, we cull a few extracts, of more particularly professional interest:

"*Present Condition of the Profession of Medicine in Spain.*—Judging from what I have seen of the medical men in Spain, I do not think them worthy of the odium and disrepute under which they suffer in other countries. It cannot be denied that they are, and have been for many years, behind the members of the profession of several other kingdoms of Europe in improvement; that they have made few discoveries in the nature and treatment of diseases; in the proper method of curing wounds, fractures, and other injuries; or in the construction of instruments and apparatus; or in chemistry, and other collateral sciences. But, still, they are not as unskilful and as illiterate as they are represented; and whether they are deservedly so or not, it is certain that they possess great respectability and influence with all classes of society. Indeed, it is not a little surprising, that, amid all the revolutions, and many disturbances which have taken place of late in the kingdom, its faculty have been permitted in a great degree to remain in a comparative state of tranquillity; and while the members of every other profession have been retrograding, and the all-powerful priesthood have been reduced to the lowest condition, that they have continued unchanged, and have maintained themselves in all their pristine prosperity. It appears to me that one of the principal causes of the disrepute in which the Spanish faculty is held by their brethren, is the very small compensation they are said to receive for their services. Their compensation, measured by a foreign standard, is truly moderate and inadequate, but in reality is not so much so as believed; for in the first place, their fees being low, they are oftener employed; and in the second they are paid in cash, so that they do not from having long accounts, and numerous charges against their patients, appear to be in the receipt of very large incomes, and yet have in fact very small ones, as is the case in the United States and other countries, where accounts are kept for professional services rendered." * * * *

"The only periodical published is in Madrid, and this has so limited a circulation that it was impossible for me to obtain a single copy. For recent information they rely almost exclusively on French publications, and chiefly on the periodicals published in Paris. The only English works I met with were those of Cullen, and other medical authors of about the same period. These works were mostly translated into Spanish. Of those by American authors I met with none; and the Spanish physicians seem to be acquainted with few of even the most celebrated of them. Finally, in consequence of the perusal of French publications, Spain having been so often overrun by their armies, and many of the Spanish faculty having been partly, if not altogether, educated in the medical schools of France, their practice appears to be chiefly that of the modern French school, though it still partakes considerably of the ancient practice in Spain and other parts of Europe."

The medical colleges appear to be on the usual footing. Professorships are filled by a *concours*. 'Bandages and Medical Jurisprudence,' we observe oddly jumbled together in a single chair at the college of Cadiz.

We have dwelt at such length upon previous points, that we cannot pause at Minorca, Marseilles, Toulon, Sicily, Malta, Corfu, the Archipelago, Palestine, Smyrna, and their "climate, animals, antiquities, curiosities, inhabitants, hospitals, bagnios, apothecaries, physicians, and diseases." The notices of these places contain interesting information of a varied character.

